# A novel mice model of acute flares in osteoarthritis elicited by intra-articular injection of cultured mast cells

**DOI:** 10.1186/s40634-021-00391-6

**Published:** 2021-09-08

**Authors:** Junpei Dan, Masashi Izumi, Hiroko Habuchi, Osami Habuchi, Shogo Takaya, Yusuke Kasai, Ryuzo Hayashi, Koji Aso, Takahiro Ushida, Masahiko Ikeuchi

**Affiliations:** 1 Department of Orthopedic Surgery, Kochi Medical School, Kochi University, 185-1 Oko-cho, Nankoku, Kochi Pref Japan; 2 Multidisciplinary Pain Center, Aichi Medical University, Nagakute, Japan; 3 Center for Innovative and Translational Medicine, Kochi University, Nankoku, Japan

**Keywords:** Mast cell, Osteoarthritis, Pain, Inflammation, Flare

## Abstract

**Purpose:**

Mast cells are multifunctional in osteoarthritis (OA), and infiltration of activated mast cells likely contributes to disease severity and progression. However, the detailed mechanisms of action are unclear. The purpose of this study was to elucidate the role of mast cell infiltration in OA at histological level using a new mice model and to investigate pharmacological inhibitory effects of existing mast cell stabilizers in this model.

**Methods:**

Mice were injected intra-articularly with monosodium iodoacetate (MIA 0.5 mg) or PBS on day 0, and PBS, with or without mast cells (MC: 1 × 10^6^ cells) on day 14. They were divided into four groups: OA flare (MIA + MC), OA (MIA + PBS), MC non-OA (PBS + MC), and PBS non-OA (PBS + PBS). In OA flare, the MC stabilizer drug (tranilast: 400 mg/kg/day) or PBS was administered intraperitoneally from days 15 to 21.

**Results:**

Histologically, modified Mankin score of the OA flare was significantly higher than that of OA (7.0 [1.8] vs. 3.3 [1.3], *P* < 0.05), and a larger number of mast cells was observed in OA flare than in OA (34.5 [6.3]/mm^2^ vs. 27.2 [2.3]/mm^2^, *P* < 0.05) on day 22. OA flare also showed acute exacerbation of pain and increased gene expression of pro-inflammatory cytokines and aggrecanase compared with OA. Administration of tranilast to OA flare-up provoked significant improvements in term of histological changes, pain, and gene expression at day 22.

**Conclusion:**

Our novel model possibly mimics OA flare conditions, which may open a new strategy of disease-modifying treatment for OA, focused on controlling the multiple functions of mast cells.

## Background

Osteoarthritis (OA) is the most common form of arthritis in the elderly and has long been considered a non-inflammatory condition characterized by progressive degeneration of articular cartilage [9]. However, a growing body of evidence has shown that local inflammation, that is, synovitis, plays an essential role in the pathophysiology of OA as it is a key predictor of joint failure and a determinant of pain [22].

Infiltration of immune cells into synovial tissue is an important phenomenon in the development and progression of OA. Although the underlying cellular and molecular mechanisms such as rheumatoid arthritis have not been fully elucidated, the most frequent types of immune cells found in OA joints are macrophages, T cells, and mast cells (MC) [16, 17]. There is limited research in the role of MCs in the pathophysiology of OA as compared to that of macrophages and T cells. Previous reports from the 1990s, however, showed that the number of MCs increased in the synovium and synovial fluid, which was associated with the degree of inflammation in OA patients [4, 10, 35]. Recently, an immunohistochemistry study demonstrated that the number of activated MCs in the synovium was positively correlated with the severity of synovitis and radiological disease progression of knee OA [8]. These reports strongly suggest that synovial MC infiltration is associated with a variety of inflammatory changes, which play a key role in the pathophysiology of OA.

Pain is a major symptom of OA, and acute aggravation of pain between chronic stable states is frequently seen in clinical practice. This is known as ‘pain flare’ or ‘flare-up’ [25]. Flares have been described as inflammatory in nature and are often prolonged for a while [25]. The underlying mechanisms of this condition, however, have not yet been fully described. Although a number of painful rodent OA models have been developed, there is inadequate basic research focusing on histological changes in the acute flare. Regarding treatment, coping strategies against the OA flare condition have not been established clinically [28]; hence, symptom-modifying drugs are often used continuously. However, adverse side effects are always problematic, especially in elderly patients [30].

In this study, we hypothesized that acute flares would develop partly because of activated MCs in the OA joint and the results of this study can help to identify a new disease-modifying approach focused on controlling the multiple functions of mast cells.

The objectives of this study were therefore:

- 1) To elucidate the role of mast cell infiltration in OA at histological level using a new mouse model mimicking the OA flare condition.
- 2) To investigate the pharmacological inhibitory effect of existing mast cell stabilizers in this model.

## Methods

### Experiment 1: Development of OA flare model

#### Animals

Male C57BL/6 mice aged 7 weeks (18–20 g, Japan SLC, Hamamatsu, Japan) were housed under standard conditions. All experiments were carried out after 1 week of acclimatization to the environment. The experimental protocol was approved by the Institutional Review Board of our University for Animal Research (M-00078).

#### Preparation of MC

Bone marrow cells were isolated from the femurs of 8-week-old male C57BL/6 mice and cultured in RPMI 1640 medium containing 10% foetal bovine serum and 50% conditioned medium of WEHI-3 cells in plastic culture dishes [32]. The culture was continued for 5 weeks with weekly changes in the medium. Cell viability was determined by trypan blue staining. The concentration of the MC solution was then adjusted to 1 × 10^6^ cells / 20 μl PBS [11].

#### Induction of OA and intra-articular injection of MC

**Fig. 1:**
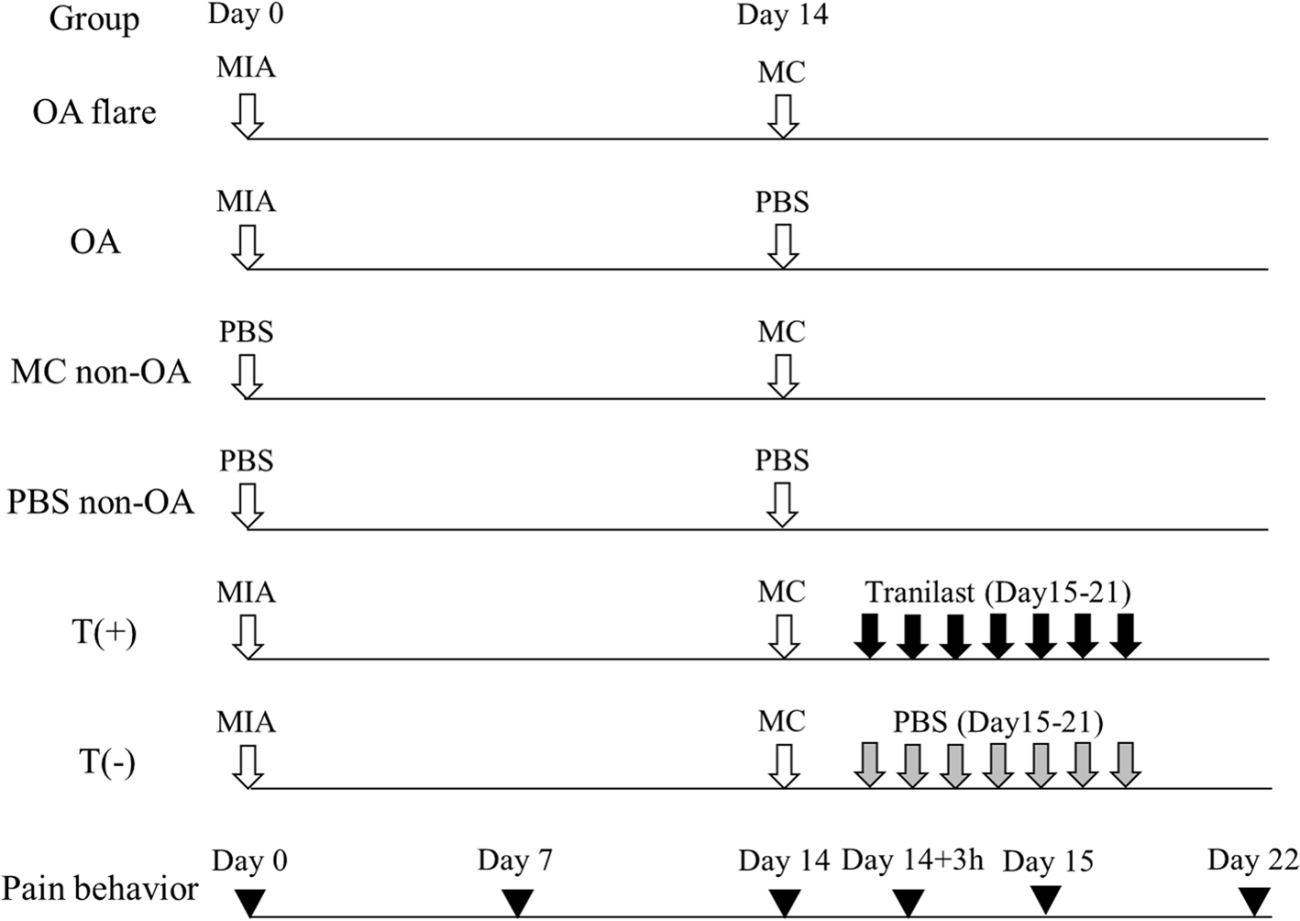
Experimental design. Mice were divided into the following 4 groups according to injected solutions at day 0 and 14; OA flare (MIA + MC), OA (MIA + PBS), MC non-OA (PBS + MC), and PBS non-OA (PBS + PBS) (experiment 1). Furthermore, in OA flare, the mice were divided into 2 groups depending on the tranilast injection (T ( +), T (-)) (experiment 2). Pain behavior was measured at specific timing of days 0, 7, 14, 14 + 3 h (3 h after injection), 15, and 22. Mice were euthanized at day 22; subsequently, histological analyses and RT-PCR were conducted

OA was induced through a single intra-articular injection of monoiodoacetate (MIA), which is a well-established model associated with cartilage degeneration, joint destruction, and persistent pain behavior [31, 34, 46]. On day 0, the mice were anaesthetized by isoflurane inhalation. After sterilising their knee with 70% ethanol, MIA (0.5 mg) dissolved in 10 μl saline was injected into the right knee through the patellar tendon using a 30G Hamilton micro syringe. PBS was used as a control. On day 14, the MC solutions (1 × 10^6^ cells / 20 μl PBS) were injected into the ipsilateral knee using the same procedure as above. The concentration of the MC solution was determined according to a previous intradermal injection of local MC injection intra-dermally [11]. PBS was injected as a control instead of MC solution. For subsequent analyses, mice were divided into four groups as follows according to the injected solutions on day 0 and day 14: OA flare (MIA + MC), OA (MIA + PBS), MC non-OA (PBS + MC), and PBS non-OA (PBS + PBS) (Fig. 1). First, two groups (MIA + MC and MIA + PBS) were created to investigate the role of MC infiltration in the OA joint. As no reports were available for MC injection into a normal joint, the MC non-OA (PBS + MC) group developed secondarily. The PBS non-OA (PBS + PBS) group was used as control.

#### Histopathological analysis

Mice were euthanized on day 22, and their knees were extracted and fixed in 4% paraformaldehyde for one day, decalcified in 13% formic acid solution for 2 weeks, and then embedded in paraffin. The specimens were sagittally sectioned at 5 μm thickness and stained with toluidine blue. Histological sections were observed using a Nikon ECLIPSE 80i microscope (Nikon, Tokyo, Japan). Histopathologic classification of the severity of OA was graded using a modified Mankin score ranging from 0 to 13 points (0 as best, 13 as worst) [47]. In addition, MCs were identified as cells with round or elliptical morphology and numerous cytoplasmic metachromatic granules [36] at the meniscosynovial junction. The number of MCs in the meniscosynovial junction was counted at 400 × magnification. The area of the meniscosynovial junction was then measured using image analysis software (Image J) [37] and the number of MCs was normalised by the area (number/ mm^2^).

#### Immunofluorescence staining

Immunofluorescence staining was performed to confirm the distribution of injected (exogenous) MC over time. Green fluorescent protein (GFP) expressing MCs were obtained from C57BL/6-Tg (CAG-EGFP) mice provided by Japan SLC (Hamamatsu, Japan) cultured for 5 weeks, and they were injected into the OA knees (MIA injected 14 days ago) using the same protocol as above. Mice were euthanized at 1, 3, and 7 days after injection of GFP-expressing MCs, and paraffin-embedded sections of the knee were obtained at each point in time. The presence of MCs, including both exogenous and endogenous cells, was investigated using mast cell protease-6 (MCP-6) immunofluorescence staining. The knees were extracted and fixed in 4% paraformaldehyde for one day, decalcified with 0.5 M EDTA for 2 weeks, and then embedded in paraffin. Following deparaffinization in xylene and washing with graded alcohol, activation with 0.1% trypsin was performed at 37 °C for 30 min. After blocking with 5% normal goat serum for GFP and 5% normal donkey serum for MCP-6, the slides were incubated at room temperature (20–25 °C) for an hour. Primary antibodies against rabbit GFP (1:500, Novus Biologicals, CO, USA) and goat MCP-6 (1:500, Santa Cruz Biotechnology, TX, USA) were applied to the sections and incubated overnight at 4° C. The next day, sections were incubated with the secondary antibody for 2 h. Goat anti-rabbit IgG Alexa Fluor 546 (1:400, Thermo Fisher Scientific, MA, USA) and donkey anti-goat IgG Alexa Fluor 488 (1:400, Thermo Fisher Scientific, MA, USA) were used as the corresponding secondary antibodies. Before, between, and after each incubation step, the sections were washed three times for 5 min PBS. Finally, all sections were mounted with Vectashield (Vector, Burlingame, CA). Sections were viewed with an FV-1000D laser confocal microscope (Olympus, Tokyo, Japan) and GFP/MCP6 immuno-reactive cells were observed.

#### Pain behavior test

Mice were individually placed in a transparent container at room temperature, and their spontaneous activities were continuously recorded using a digital camera (Nikon, Tokyo, Japan) for 10 min. Two observers independently watched the recordings and provided a semi-quantitative evaluation of pain behavior using Stance score [12] as follows: 0 = No visible impairment of gait or stance, the foot firmly placed flat on the surface with normal spread of toes. 1 = Moderate impairment of stance, the foot is placed on the ground with toes tightly contracted. 2 = Severe impairment of gait and stance; foot either entirely elevated from the ground or only the lateral part of the foot lightly touching the ground and toes tightly pulled together. As the ‘stance’ varied frequently during the observation period, the highest score maintained for at least 10 s was therefore assigned as the final score. Intermediate scores (0.5 and 1.5) were used for animals that displayed a behavior in between the definitions described above. Scoring was performed on days 0, 7, 14, 14 + 3 h (3 h after injection), 15, and 22 (Fig. 1). The average score from the two observers was used for data analyses. Ipsilateral paw hyperalgesia was assessed simultaneously. After 30 min of acclimatisation, paw withdrawal threshold was evaluated using von Frey filaments (North Coast Medical Inc., Morgan Hill, CA, USA) 1.65, 2.36, 2.44, 2.83, 3.22, 3.61, 3.84, 4.08, 4.17, and 4,31 (corresponding to 0.008, 0.02, 0.04, 0.07, 0.16, 0.4, 0.6, 1.0, 1.4, and 2.0 g, respectively).

#### Real time-polymerase chain reaction (RT-PCR) analysis

Total RNA was isolated from the right side of the infrapatellar fat pad on day 22, and mRNA levels of IL-1β, TNF-α, IL-6, ADAMTS-4, and NGF was measured by the quantitative RT-PCR intercalated method. cDNA was synthesised using the ReverTra Ace® qPCR RT Kit (Toyobo, Osaka, Japan). Reaction mixtures for RT-PCR were composed of 2 μl cDNA, 1.6 μM specific primer pair, 10 μl TB Green Premix Ex Taq II (Takara, Kyoto, Japan), 0.4 μl ROX reference dye, and 6 μl nuclease free water in a final volume of 20 μl. Quantitative PCR was performed using a real-time PCR system (StepOnePlus; Thermo Fisher Scientific, MA, USA) to detect the relative mRNA expression levels. The PCR cycle protocol was as follows: the holding stage performed one cycle at 95 °C for 30 s, the cycling stage was repeated 40 times at 95 °C for 15 s, and at 60 °C for 60 s. mRNA expression was normalised to the levels of HPRT mRNA.

### Experiment 2: Treatment of OA flare model with tranilast

To investigate the possibility of pharmacological suppression of MC-injection-induced acute flare, tranilast, an established MC stabilizer drug, was systemically administered to OA flare mice. This is according to a previous report using a collagen-induced arthritis model mimicking RA [14]. Tranilast has been used therapeutically for many years as an anti-allergic drug against bronchial asthma, allergic rhinitis, atopic dermatitis, and hypertrophic scars [9], and has been approved by the National Insurance System in our country. In the OA flare group, 1 ml of tranilast (400 mg/kg/day) or PBS was injected intra-peritoneally with a 27G syringe once a day from day 15 to 21. According to previous studies, tranilast was administered orally [40] or through an intraperitoneal injection [14], yet no study has been conducted using a local injection. In this study, we selected intraperitoneal injections to avoid differences in tranilast intake by oral administration. The mice were divided into two groups depending on the tranilast injection: the T ( +) and T (-) groups. To evaluate the pharmacological effects of tranilast, histology (modified Mankin score and number of MCs), pain behavior (stance score), and RT-PCR (IL-1β, TNF-α, IL-6, ADAMTS-4, and NGF) were examined in an identical manner to the first experiment. An overview of the experimental protocol is presented in Fig. 1.

#### Statistical analysis

The primary outcome of this study was histological changes in the OA flare model compared with the OA model in Experiment 1, and the pharmacological inhibitory effects of tranilast against this altered histology in Experiment 2. Pain behavior and joint inflammation were set as secondary outcomes in both experiments. Our sample size was determined by referring to a previous study that examined the modified Mankin score in a mouse MIA OA model. In the study, it was found that a 20% decrease in this histological score after a specific treatment was statistically significant [1]. Calculations were performed according to this difference with 90% statistical power and α level of 0.05, and it was determined that at least 10 mice were needed for OA flare and OA group in Experiment 1 and, T ( +) and T (-) group in Experiment 2. Finally, a sample size of 13 mice were chosen to allow for possible dropouts. Kruskal–Wallis test followed by Mann–Whitney U test with Bonferroni correction was used to compare pain behavior, modified Mankin score, and MC number among the four groups (OA flare, OA, MC non-OA, and PBS non-OA). The Mann–Whitney U test was used to compare pain behavior, modified Mankin score, and MC number with or without tranilast administration to the OA flare mice (T ( +) vs. T (-) groups). To evaluate mRNA gene expression levels between the groups (OA flare vs. OA group, and T ( +) vs. T (-) group), the Mann–Whitney U test was also used. Statistical significance was set at *p* < 0.05. All statistical analyses were performed using SPSS software (ver. 26.0; SPSS, Chicago, IL, USA).

## Results

### Experiment 1

**Fig. 2:**
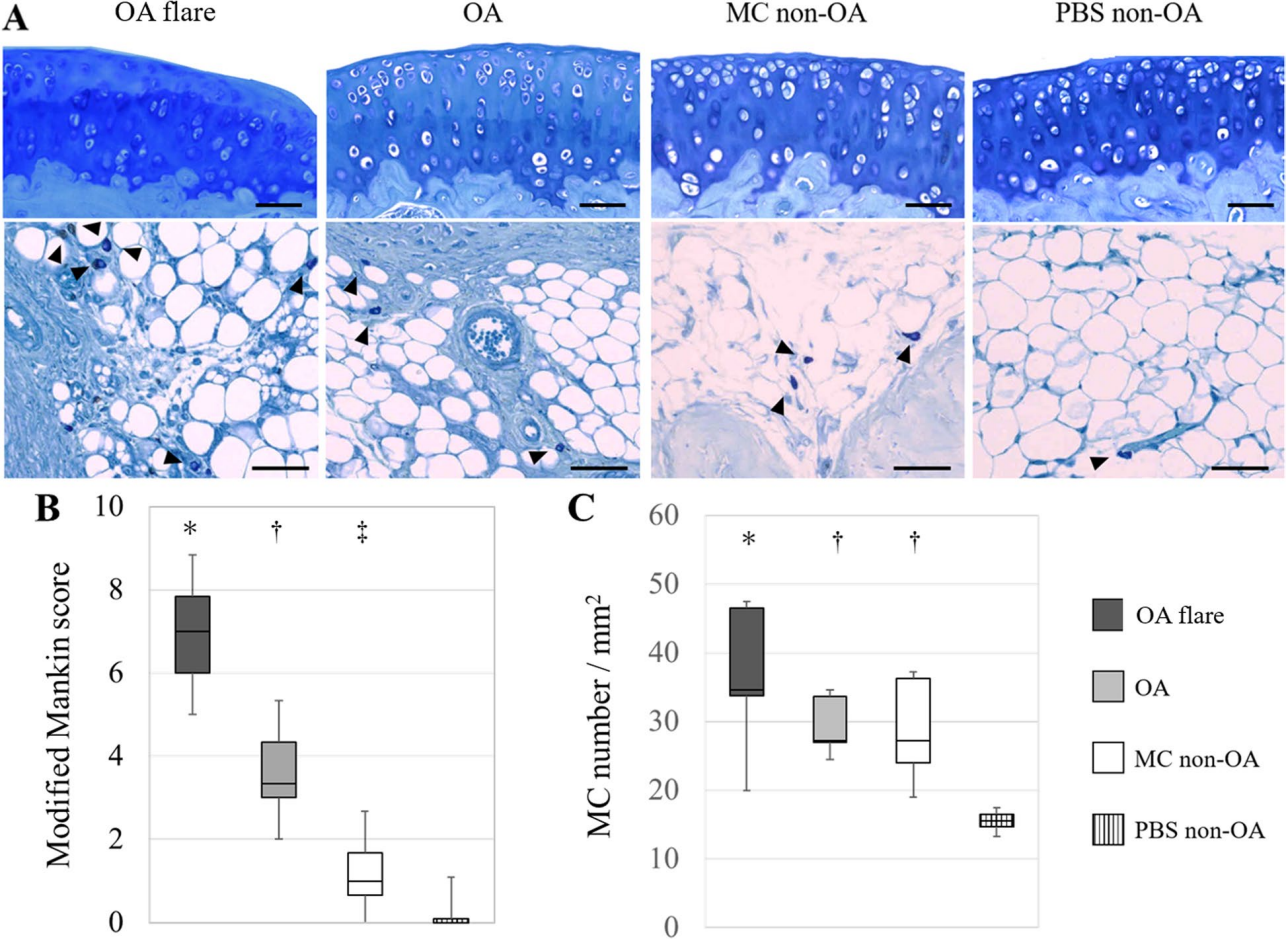
**A** Histopathological changes of OA and MC infiltration (toluidine blue staining). Arrowhead indicates MC. **B** Modified Mankin score (Median [interquartile range]). OA flare (n = 13), OA (n = 13), MC non-OA (n = 16), and PBS non-OA (n = 4). *: *P* < 0.05 compared with OA, MC non-OA, and PBS non-OA; †: *P* < 0.05 compared with MC non-OA and PBS non-OA; ‡: *P* < 0.05 compared with PBS non-OA. **C** Number of MC located in the synovium around the meniscosynovial junction. (Median [interquartile range]). *: *P* < 0.05 compared with OA, MC non-OA, and PBS non-OA; †: *P* < 0.05 compared with PBS non-OA. (Black bar: 50 μm)

**Fig. 3:**
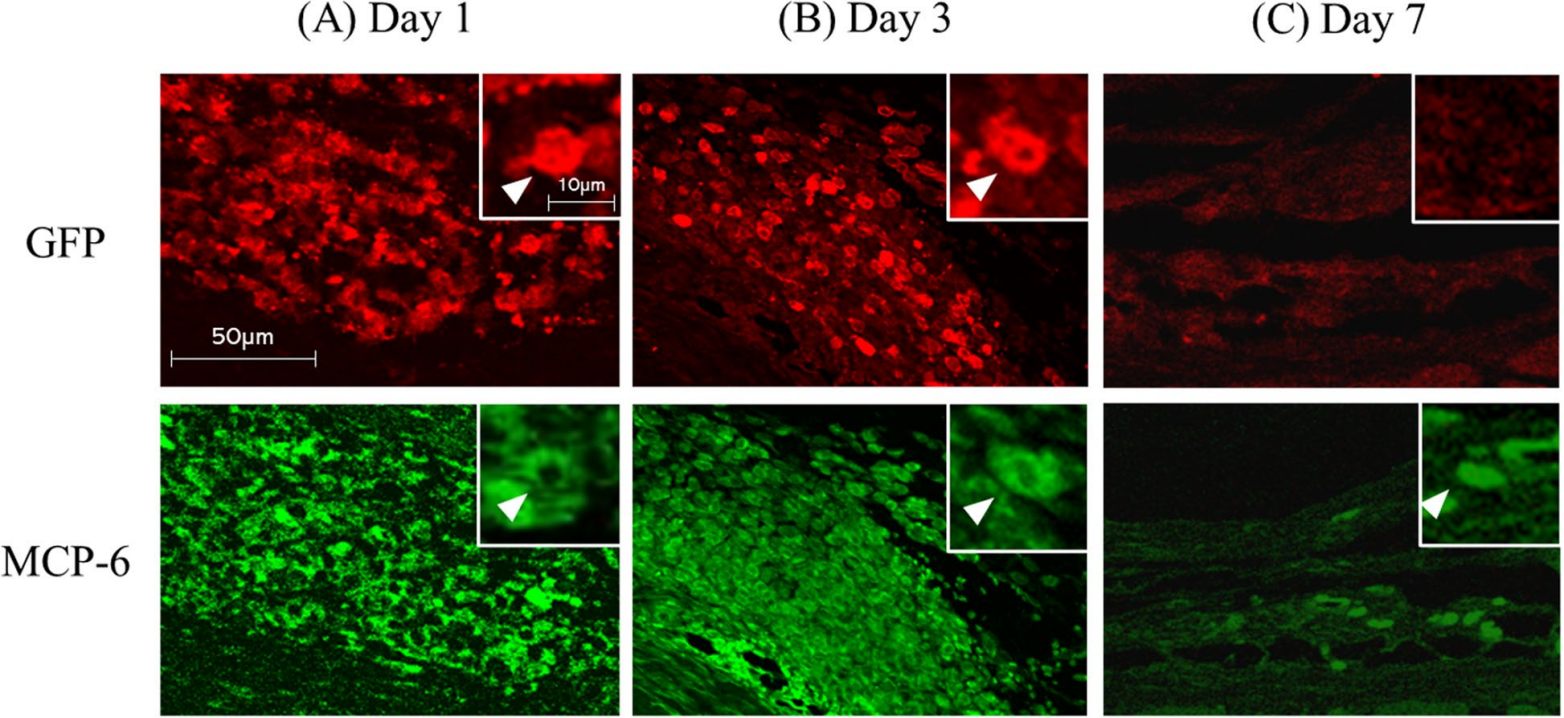
Representative photos presenting distributions of injected MC in the knee joint over time. Immunofluorescence staining (**A**) 1 day, (**B**) 3 days, and (**C**) 7 days after the MC injection. Cells labelled with GFP and MCP-6 were observed in (**A**) and (**B**), which demonstrated that exogenous MC existed at that time. MCP-6 positive cells still existed in (**C**), but GFP positive cells were not observed, suggesting that endogenous MC accounted for the majority 7 days after the MC injection. Magnification was identical in all 6 panels

**Fig. 4:**
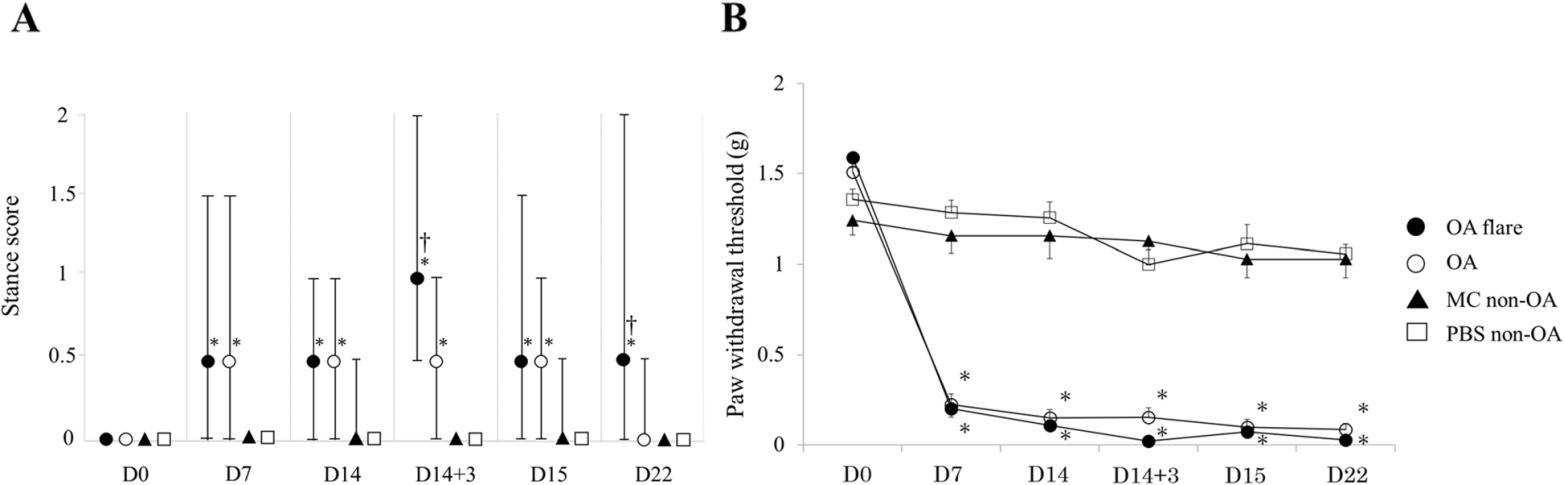
**A** Median (range) of Stance score in each group at day 0 before the first injection (D0), day 7 (D7), day 14 before the second injection (D14), 3 h after the second injection (D14 + 3), day 15 (D15), and day 22 (D22). **B** Mean (SEM) of ipsilateral paw withdrawal threshold in each group at D0, D7, D14, D14 + 3, D15, and D22. OA flare (filled circle, n = 31), OA (open circle, n = 30), MC non-OA (triangle, n = 12), and PBS non-OA (square, n = 9), respectively. *: *P* < 0.05 compared with PBS non-OA; †: *P* < 0.05 compared with OA

**Fig. 5:**
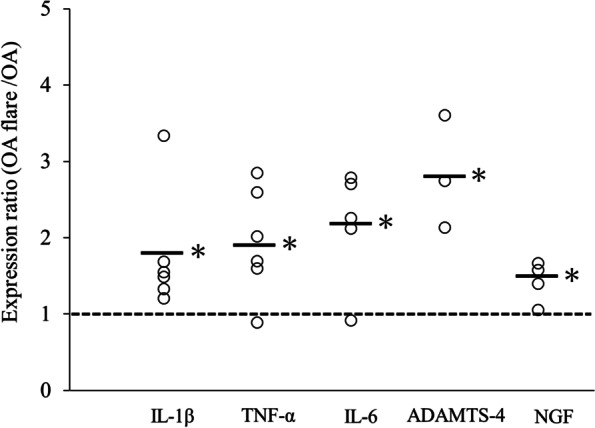
Levels of mRNA expression ratio (OA flare divided by OA) of IL-1β, TNF-α, IL-6, ADAMTS-4, and NGF. Each plotted circle contains infrapatellar fat pad from three mice. (Bar: median *: *P* < 0.05 compared with OA)

A sufficient amount of viable MC was successfully obtained after five weeks of culture. Histologically, the modified Mankin score (median [interquartile range]) was 7.0 [1.8] in the OA flare group, which was significantly higher than 3.3 [1.3] in the OA group (*P* < 0.05; Fig. 2). The number of MCs (median [interquartile range]) also showed a significant difference among the groups (OA flare 34.5 [6.3] /mm^2^ vs. OA 27.2 [2.3] /mm^2^, *P* < 0.05; Fig. 2). Immunofluorescence staining (A) 1 day, (B) 3 days, and (C) 7 days after MC injection showed that MC labelled with GFP and MCP-6 was observed in (A) and (B), which demonstrated that exogenous MC existed at that time. MCP-6 positive cells were still present in (C), but GFP-positive cells were not observed, suggesting that endogenous MC accounted for the majority 7 days after MC injection (Fig. 3). MIA injection induced significant pain and paw hyperalgesia compared to PBS injection (*P* < 0.05; Fig. 4). After the second injection at day 14, Stance score (median (range)) was 1.0 (0.5–2.0) in the OA flare group which was significantly higher than 0.5 (0–1.0) in the OA group (*P* < 0.05; Fig. 4). However, this difference was not detected in von Frey test. The MC non-OA group showed no pain and paw hyperalgesia, which was comparable to the PBS non-OA group (Fig. 4). The mRNA expression levels of IL-1β, TNF-α, IL-6, ADAMTS-4, and NGF were 1.5–2.8 times higher in the OA flare group than in the OA group (*P* < 0.05; Fig. 5).

### Experiment 2

**Fig. 6:**
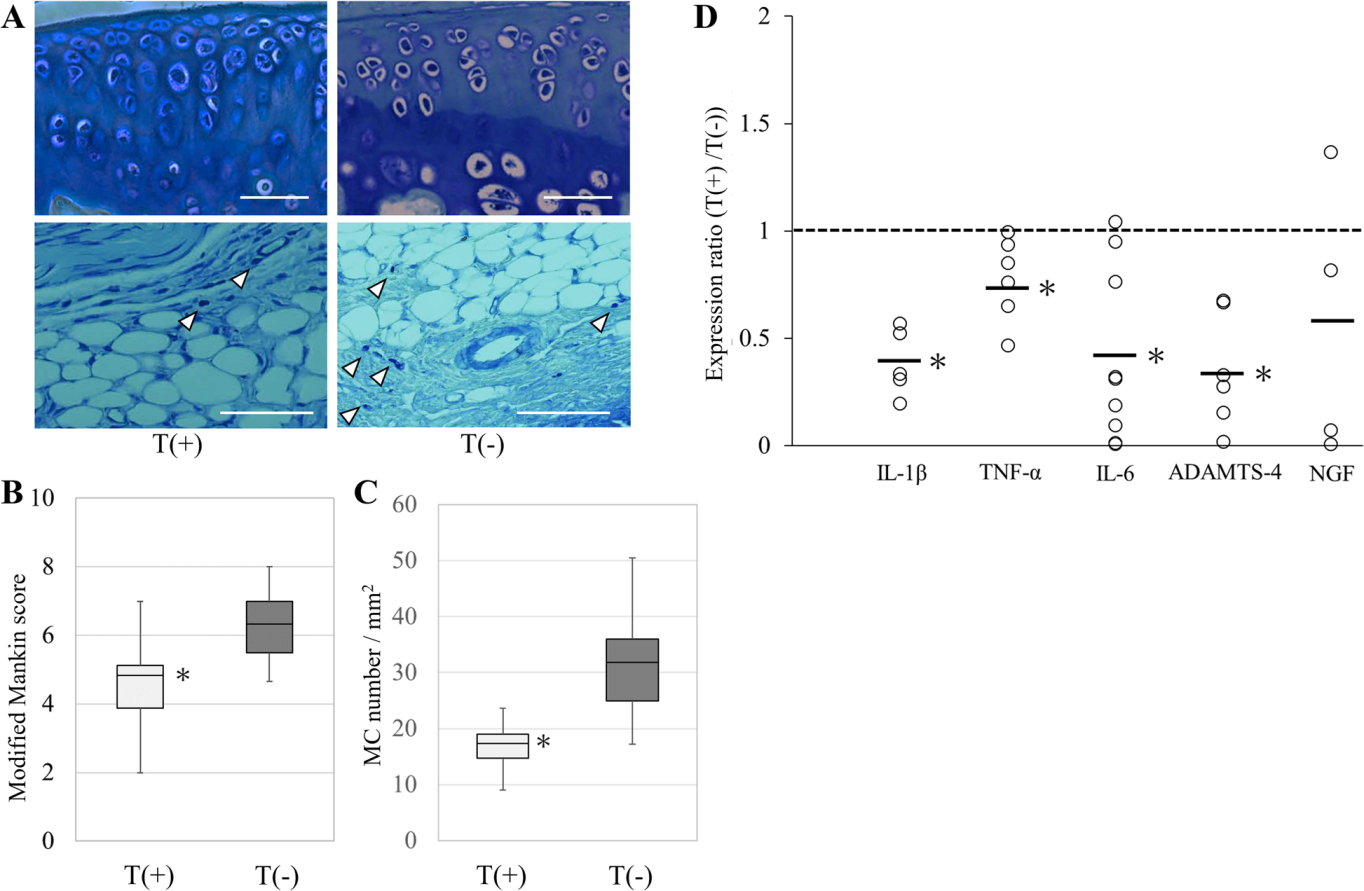
**A** Effect of Tranilast on articular cartilage degeneration and MC infiltration in the synovium around the meniscosynovial junction. Arrowhead indicated MC. **B** Modified Mankin score (Median [interquartile range]). **C** Number of MC located in the synovium around the meniscosynovial junction. (Median [interquartile range]). T ( +) group (n = 13), T (-) group (n = 13). *: *P* < 0.05 compared with T (-) group (white bar: 50 μm). **D** Levels of mRNA expression ratio (T ( +) divided by T (-)) of IL-1β, TNF-α, IL-6, ADAMTS-4, and NGF. Each plotted circle contains an infrapatellar fat pads from three mice (bar: median *: *P* < 0.05 compared with T (-) group)

Histologically, the modified Mankin score was 4.8 [1.2] in the T ( +) group, which was significantly lower than 6.3 [1.5] in the T (-) group (*P* < 0.05; Fig. 6). The number of MCs also showed a significant difference among the groups (T ( +) 17.3 [4.2] /mm^2^ vs. T (-) 31.8 [11] /mm^2^, *P* < 0.05; Fig. 6). Regarding the pain behavior, both T ( +) and T (-) groups showed similar pain on day 15 with the Stance score of 1.0 (0.5–1.5) in T ( +) group and 1.0 (0.5–2.0) in T (-) group (*P* = 0.54). On day 22, the stance score was reversed to 0.5 (0–0.5) in the T ( +) group following repeated tranilast injections; however, this change was not observed in the T (-) group (*P* < 0.05). The mRNA expression levels of IL-1β, TNF-α, IL-6, ADAMTS-4 were 0.35–0.75 times lower in the T ( +) group than in the T (-) group (*P* < 0.05; Fig. 6).

## Discussion

This study focuses on the role of MC infiltration in the OA joint to clarify some of the underlying mechanisms of acute OA flare conditions. In clinical practice, symptom-modifying drugs are generally used for this condition; however, they often have limited effects on disease progression. In addition, adverse events associated with the continuous use of this drug are not rare, especially in elderly patients. The results of this study can help to identify a new disease-modifying approach focused on controlling multiple function of MC. Our novel OA flare mice model showed articular cartilage degeneration together with MC infiltration at histological level, acute exacerbation of pain behavior, and increased gene expression of proinflammatory cytokines and aggrecanase in the knee joint compared to the conventional MIA OA mice model. Furthermore, systemic administration of tranilast in the OA flare model significantly suppressed histological deterioration, pain behavior, and gene expression associated with disease progression compared with the no-treatment group.

### Role of MC in osteoarthritic joint

In OA joints, synovial MCs secrete histamine and serotonin by degranulation, which causes fibroblast proliferation and upregulates vascular permeability and neovascularization [29]. Tryptase is also released by degranulation, which is linked to cartilage matrix degradation, leukocyte recruitment, and fibroblast activation [29, 45]. Metabolites of arachidonic acid upregulate the chemotaxis of neutrophils and leukocytes, which are involved in the development of inflammation and pain. They also enhance the production of MMPs and ADAMTSs from chondrocytes, which induces further cartilage damage [3, 29]. Proinflammatory cytokines and chemokines activate other immune cells that cause inflammation and pain [45], and induce the release of MMPs and ADAMTSs to process articular cartilage catabolism [43]. Although not all the mechanisms of action above were investigated in the current study, intraarticular injection of cultured MCs into the established OA models significantly elicited histological deterioration, pain aggravation, and elevation of IL-1β, TNF-α, IL-6, and ADAMTS-4, which partly mimics the status of acute flares and subsequent reactions in OA joints.

Recently, NGF has become particularly interesting because some clinical studies have demonstrated that injection of a monoclonal antibody against NGF provided persistent pain relief and improved function in moderate to severe OA [18, 26]. NGF affects many pain-related molecules by signal transduction through TrkA and p75 receptors expressed on nociceptors [15, 20]. Under inflammatory conditions, NGF induces proinflammatory cytokine production from immune cells, and the synthesised cytokines further upregulate NGF expression [42]. Among the immune cells, MC plays an essential role because it can aggressively produce NGF by itself and also respond to NGF through the expression of TrkA [23]. In this study, MC injection into OA joints significantly enhanced local NGF expression and pain behavior, which possibly reinforce the rationale of this theory.

### MC engraftment into arthritis model

Previously, Lee et. al reported that a mouse model that genetically lacks MCs does not cause K/BxN serum transfer-induced ankle arthritis and histological deterioration in the joints, but after systemically engrafting MC, arthritis was observed [19]. Wang et. al showed that genetic mouse models of mast cell deficiency attenuated OA pathology induced by destabilisation of the medial meniscus, and transfusion of bone marrow-derived mast cells into these mice systemically and intra-articularly reversed the relative protection conferred by MC deficiency [48]. These reports strongly support that MC plays a key role in the pathophysiology of OA. In this study, injection of exogenous MC to MC-deficient mice, which are often produced by mutation of the c-kit gene, may provide clearer results regarding the role of exogenous MC [11]. However, the use of MC-deficient mice in our model appears to be not necessarily suitable for estimating the role of endogenous MC, since c-kit is expressed in various hematopoietic stem cells and bone marrow precursor cells and, consequently, mice with a mutation in the c-kit gene potentially exhibit abnormalities in the differentiation of various immune cells other than MC [28]. In this regard, our novel approach without any gene mutation may be more suitable for mimicking facilitated MC infiltration in clinical situations.

### Relationship between exogenously engrafted MC and endogenous MC in OA flare model

Very few studies have described the fate of exogenously engrafted MCs in rodents. Nakano et al. injected MC intra-peritoneally into MC-knockout mice and confirmed viable MCs after a week [27], which was inconsistent with our immunohistochemistry results. The main reason for this discrepancy may be explained by genetic modification of animals, that is, MC-knockout or normal mice, as mentioned above. In our OA flare model, exogenous MC was mainly observed at first, but endogenous MC accounted for the majority 7 days after MC injection. This finding was unexpected but highly interesting because both exogenous and endogenous MC possibly collaborated and contributed to the pathophysiology of this model. The plausible mechanism of action is as follows: Since MC injection did not work at all in the normal joints, degranulation of exogenous MC probably played a key role in OA joints and subsequently secreted proinflammatory mediators might contribute to trigger acute flares. Additionally, these mediators and various growth factors also contribute to MC chemotactic migration [6]. Therefore, the effect of exogenous MC would not continue for 7 days, but several mediators released from exogenous MC might contribute to the recruitment and activation of endogenous MC that would also work subsequently. Our OA flare model seems to have a potential advantage in investigating the molecular mechanisms through which pain is elicited by MCs in OA. However, the extent to which endogenous MC, which is presumed to participate in the elicitation of pain in clinical status, contributed to the results could not be adequately estimated in this model.

### Effects of MC stabilizer

As described, MC stabilizers seem to be a promising treatment option for acute flares in OA. Blockages of histamine, a representative molecule secreted from MC, was associated with a reduction in knee OA prevalence in a large observational study [41]. Tranilast is a membrane stabilizer drug that inhibits the release of various mediators from MCs. In addition to anti-allergic effects that suppress the degranulation of histamine release, tranilast inhibits the expression of many cytokines, such as IL-6 and TNF-α [14, 33, 40], chemokines [2, 5] and proteases [38, 39]. Recent animal and human studies have also reported the efficacy and safety of MC stabilizer drugs, such as tranilast and ketotifen. Systemic administration of tranilast in a mouse collagen-induced arthritis model mimicking RA suppressed histological joint destruction and production of inflammatory cytokines and metalloproteases [14, 40]. In a joint contracture model after trauma, ketotifen significantly decreased the number of myofibroblasts and MC in the affected joint capsule, which was associated with an improvement in contracture [24]. Furthermore, ketotifen mixed with a posttraumatic human elbow joint capsule led to decreased collagen gel contraction in vitro [13]. Consistent with these reports, our novel OA flare model demonstrated for the first time that histological OA changes, pain behavior, and gene expression of local inflammatory cytokines and aggrecanase were successfully suppressed by systemic administration of tranilast. Since the number of MCs in the synovium significantly decreased and MC-induced pain behavior was reversed in the T ( +) group, a possible role of tranilast seemed to reduce the degranulation of the exogenous MC, which might subsequently attenuate the activity of endogenous MC.

There are many established drugs for RA that can induce remission of the disease. However, the pathophysiology of OA remains unclear; therefore, no disease-modifying drugs are clinically available. In this regard, tranilast is a promising candidate as a disease-modifying therapeutic drug for OA. This is because it has been used therapeutically for many years as an anti-allergic drug against a bronchial asthma, allergic rhinitis, atopic dermatitis and hypertrophic scars [44], which were all approved by the National Insurance System of our country. Of course, there are some hurdles to overcome, for example, long-lasting effects and possible adverse events in clinical trials. However, it seems to be a much easier way to reach our ultimate goal when compared to establishing new drugs.

### Limitations

This study had some limitations. First, the MIA OA was a chemically induced model so that the results may not be identical to mechanically induced OA such as a meniscus resection model. Second, the contribution of other immune cells, such as macrophages and T cells, was not independently evaluated in this model. As the interaction of immune cells in OA pathology has received a lot of attention [7], that further studies focusing on this topic are warranted. Third, the degranulation of MCs in the OA flare model was not directly confirmed by immunohistochemistry. However, according to the literature, degranulation plays a key role in the immediate reaction of injected MC [21], which was also supported by our results of the MC non-OA model that showed no response to MC injection. Finally, the effects of MC injection and tranilast administration on day 22 were not investigated. Further studies are warranted to elucidate the long-term advantages of tranilast in OA progression and persistent pain.

## Conclusions

Our novel OA flare mice model showed articular cartilage degeneration, together with MC infiltration at histological level, acute exacerbation of pain behavior, and increased gene expression associated with disease progression compared to the conventional MIA OA mice model. Administration of tranilast to OA flare-up provoked significant improvements in term of histological changes, pain, and gene expression. This model possibly opens a new strategy for disease-modifying treatment of OA focused on controlling the multiple functions of MC.

## Data Availability

The data that support the findings of this study are available from the corresponding author, MI, upon reasonable request.

## Abbreviations

OA: Osteoarthritis
MC: Mast cell
MIA: Mono-iodoacetate
PBS: Phosphate buffered saline
BMMC: Bone marrow-derived mast cell
IL-1β: Interleukin-1β
TNF-α: Tumor necrosis factor-α
IL-6: Interleukin-6
ADAMTS-4: A disintegrin and metalloproteinase with thrombospondin motifs-4
NGF: Nerve growth factor
RPMI 1640 medium: Roswell park memorial institute 1640 medium
GFP: Green fluorescent protein
MCP-6: Mast cell protease-6
mRNA: Messenger ribonucleic acid
RT-PCR: Real time-polymerase chain reaction
cDNA: Complementary deoxyribonucleic acid
HPRT: Hypoxanthine phosphoribosyltransferase
RA: Rheumatoid arthritis
MMP: Matrix metalloprotease
TrkA: Tropomyosin receptor kinase A
SP: Substance P
CGRP: Calcitonin gene-related peptide
